# Health‐Care Burden of Acute Exacerbation of Chronic Obstructive Pulmonary Disease in Hong Kong in the Post‐COVID Pandemic Era—Impact of Phenotype and Exacerbation History

**DOI:** 10.1002/puh2.70425

**Published:** 2026-10-27

**Authors:** Wang Chun Kwok, Ali Choo, Lu Zhou, Ting Fung Ma, Isaac Sze Him Leung, Chun Ka Wong, James Chung Man Ho

**Affiliations:** ^1^ Division of Respiratory Medicine Department of Medicine School of Clinical Medicine LKS Faculty of Medicine The University of Hong Kong Hong Kong SAR China; ^2^ Division of Cardiology Department of Medicine School of Clinical Medicine LKS Faculty of Medicine The University of Hong Kong Hong Kong SAR China; ^3^ Department of Statistics University of South Carolina Columbia South Carolina USA; ^4^ Department of Statistics The Chinese University of Hong Kong, Shatin, New Territories Hong Kong SAR China

**Keywords:** chronic obstructive pulmonary disease (COPD), COPD exacerbation, health economic, health‐care burden, post‐COVID

## Abstract

**Rationale:**

Chronic obstructive pulmonary disease (COPD) is known to be associated with substantial health‐care utilization and economic costs. Since the COVID‐19 pandemic, the epidemiology of various diseases has undergone significant changes, it is important to evaluate the health‐care impact and economic burden of acute exacerbations of COPD (AECOPD) in the post‐pandemic period, along with factors influencing this burden, to inform effective management strategies and health‐care planning.

**Objectives:**

To assess the health‐care and economic burden of severe AECOPD from 2022 to 2024, and to identify factors associated with direct health‐care costs.

**Methods:**

A territory‐wide retrospective cohort study was conducted. Adult patients with AECOPD admitted to acute public hospitals in Hong Kong were included. The primary outcomes were the total number of AECOPD admissions and the associated direct health‐care costs.

**Measurements and Main Results:**

From 2022 to 2024, a total of 11,465 patients were admitted to acute public hospitals for severe AECOPD, resulting in 25,053 hospitalization episodes and 150,717 bed days. The estimated direct health‐care costs totaled 1.259 billion Hong Kong dollars (HKD). AECOPD admission numbers increased significantly over the 3 years. Patients with eosinophilic and non‐eosinophilic phenotypes had similar annual direct health‐care costs related to severe AECOPD. Patients with co‐existing bronchiectasis had a significantly higher annual number of severe AECOPD episodes and direct health‐care costs from severe AECOPD.

**Conclusions:**

Severe AECOPD imposes a substantial burden on the health‐care system. The presence of bronchiectasis and a greater frequency of severe AECOPD episodes were identified as possible factors linked to increased direct health‐care costs.

## Graphical abstract

There was an increase in the AECOPD admission numbers after the COVID‐19 pandemic, resulting in increase in health economic burden.

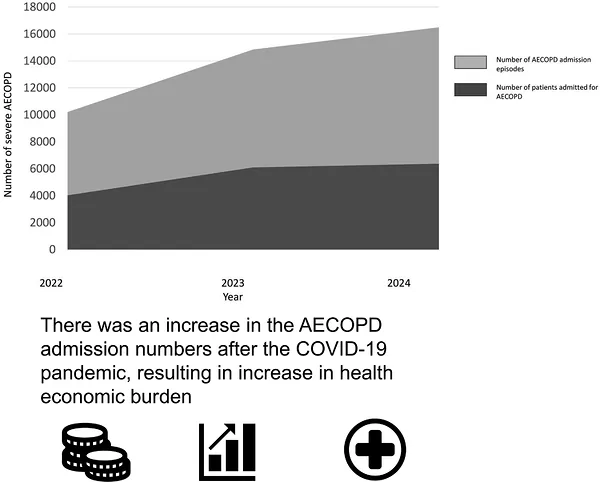

## 1 Introduction

Chronic obstructive pulmonary disease (COPD) affected 480 million people globally as in 2020 [1]. In Hong Kong, COPD also poses a considerable challenge to the health‐care system. According to the Population Health Survey 2020–22, 0.5% of non‐institutionalized persons aged 15 and above self‐reported having doctor‐diagnosed COPD [2]. Additionally, a local study reported that in 2014, there were 9613 patients diagnosed with COPD and 19,771 hospital admissions related to the disease [3].

COPD contributes significantly to both health‐care utilization and economic burden, particularly for patients with moderate to very severe disease [4]. In European countries, the estimated annual direct cost per patient from 2010 to 2019 varied across nations, with values such as €10,701 (Hong Kong dollars [HKD] 98,319.20) in Norway, €9580 (HKD 88,256) in Denmark, €7847 (HKD 72,148.40) in Germany, €7448 (HKD 68,569.60) in Italy, €7045 (HKD 64,894) in Sweden, €2896 (HKD 26,675.20) in Greece, €1963 (HKD 18,055.60) in Belgium, and €2047 (HKD 18,858.40) in Serbia [5]. A modeling study further projected that the global economic cost of COPD could reach approximately INT$4.326 trillion (HKD 45.45 trillion) during 2020–50, with an uncertainty interval of 3.327–5.516 trillion (at constant 2017 prices). This economic burden of COPD is equivalent to an annual tax of approximately 0.111% (ranging from 0.085% to 0.141%) on the global gross domestic product (GDP). China and the United States bear the largest shares of this burden, with estimated costs of INT$1363 trillion (HKD 14.35 trillion) (uncertainty interval 1034–1801) and INT$1037 trillion (HKD 10.92 trillion) (0868–1175), respectively [6]. It is also projected that in the United States, the 20‐year discounted direct medical costs attributable to COPD will amount to approximately $800.90 billion from 2019 to 2038 [7]. Although COPD is known to impose substantial health‐care costs, understanding the factors that contribute most significantly to these costs is crucial. Reported determinants of rising COPD management costs include late diagnosis, higher disease severity, more frequent acute exacerbations of COPD (AECOPD), hospital readmissions, poor treatment adherence, and exposure to risk factors for COPD [5]. Specifically, Medicare patients who experienced recent moderate or severe exacerbations or ≥2 AECOPD per year are at a higher risk of future AECOPD and tend to incur greater all‐cause health‐care costs [8].

In 2020 and 2021, the COVID‐19 pandemic led to notable shifts in the epidemiology of various diseases, including COPD. Notably, there was a significant decrease in the number of hospitalizations among COPD patients during this period, likely influenced by factors such as public health measures, social distancing, and changes in health‐care‐seeking behavior [9, 10].

Therefore, it is essential to reassess the health economic burden of COPD, particularly focusing on the direct health‐care costs linked to severe AECOPD in the post‐COVID era. Evaluating the various factors that influence health‐care costs can also offer valuable insights for clinicians, helping to identify opportunities for personalized therapy. The factors to be investigated include COPD phenotypes and comorbidities, which are increasingly recognized in recent years. For phenotypes, stratification by blood eosinophil count (BEC) is the most well recognized with prognostic implications [11]. For co‐morbidities, co‐existing bronchiectasis has also been reported to carry major clinical implications [12]. Such tailored approaches may improve patient outcomes while simultaneously optimizing health economic benefits.

## 2 Methods

A territory‐wide retrospective study was conducted to evaluate the burden of severe AECOPD from 2022 to 2024 and to identify factors associated with increased direct health‐care costs in Hong Kong. The study included adult patients admitted to acute medical units for severe AECOPD managed by the Hong Kong Hospital Authority (HKHA) during this period. Electronic health records from the Clinical Data Analysis and Reporting System (CDARS), managed by HKHA, were utilized. HKHA is a public health‐care provider that operates 43 hospitals and institutions along with 122 outpatient clinics, serving more than 90% of the Hong Kong population since 1993 [13]. CDARS captures clinical parameters of each public health‐care service user, including comprehensive medical information, including diagnoses, drug prescription records, demographics, hospital admissions, medical procedures, and laboratory results. It has been used to conduct high‐quality epidemiological studies [14, 15]. The diagnosis of COPD within CDARS has been validated, with a positive predictive value (PPV) of 81.5% (95% confidence interval: 76.1%–86.9%), ensuring reliable identification of COPD cases within the database [16].

The inclusion criteria consisted of adult patients aged ≥40 years with a principal diagnosis of COPD at the time of admission to acute hospital, identified by the International Classification of Diseases, ninth Revision (ICD‐9) code 496 in the inpatient analysis module of CDARS for hospitalizations from January 1, 2022 to December 31, 2024. This indicated that the patients were hospitalized in acute hospitals managed under HKHA with a principal diagnosis of COPD. Patients with a co‐existing asthma, as indicated by ICD‐9 code of 493, were excluded. Demographic data and clinical characteristics in the year prior to the index admission were obtained from CDARS. These included the number of prior AECOPD episodes in the 1‐year period preceding the study period, Charlson comorbidity index (CCI), baseline BEC at clinically stable state, and medications used for COPD. Clinically stable state was defined as at least 30 days away from AECOPD or antibiotics/systemic corticosteroid use. The study received approval from the Institutional Review Board of the University of Hong Kong and the Hospital Authority Hong Kong West Cluster (IRB/REC Reference No. UW 23‐356). Patient informed consent was waived for this retrospective study without active patient recruitment, and all clinical data were de‐identified.

The severity of AECOPD was determined per GOLD Recommendations, with severe AECOPD defined as one requiring emergency department visit or hospitalization [17].

The baseline BEC was defined as the highest BEC level during clinically stable state. Bronchiectasis was identified by ICD‐9 code 494. Regular use of long‐acting beta‐agonists (LABAs), long‐acting anti‐muscarinic agents (LAMAs), inhaled corticosteroid (ICS), theophylline and roflumilast was defined as continuous use of these drugs for at least 1 year prior to recruitment.

The direct health‐care costs were estimated from the total costs attributed to emergency department visits and medical ward admissions and high dependency/intensive care unit (HDU/ICU) admissions. The relevant daily costs were retrieved from the Hospital Authority Statistical Report [18]. To determine the direct health‐care costs from severe AECOPD, the calculation was done as follows: Costs for emergency department visit + (length of stay in medical ward × daily cost for medical ward) + (length of stay in HDU/ICU × daily cost for HDU/ICU).

The primary outcome was to determine the total number of patients and hospital admissions related to severe AECOPD, along with the associated direct health‐care costs. The secondary outcomes were to identify factors associated with an increase in direct health‐care costs, including COPD phenotype and the presence of comorbidities such as bronchiectasis.

### 2.1 Statistical Analysis

Categorical variables were presented as frequencies and compared using Chi‐squared tests or Fisher's exact tests, as appropriate. Continuous variables were expressed as mean ± standard deviation (SD) or median (25th–75th percentile) and compared using Student's *t*‐test or Mann–Whitney *U* tests, as appropriate. The number of severe AECOPD hospitalizations over the 3‐year period was compared using one‐way ANOVA. The relationship between disease factors and direct health‐care costs was assessed using Pearson's correlation coefficients. Linear regression was performed to assess the relationship between phenotype, annual AECOPD frequency, and burden of AECOPD. Multivariable analyses adjusting for age, sex, COPD medication use (LABAs, LAMAs, ICS, theophylline, and roflumilast), severe AECOPD in the 1‐year period and CCI were performed. Two‐sided tests were conducted, with statistical significance set at *p* < 0.05. All statistical analyses were performed using SPSS version 29.

## 3 Results

There were 11,465 COPD patients admitted to acute medical hospitals for severe AECOPD from 2022 to 2024, accounting for 25,053 hospital admissions and 150,717 bed days. The total direct health‐care costs attributed to these severe AECOPD were HKD 1.259 billion. Within the study period, 3624 patients died. The cohort consisted of 10,421 (90.9%) male patients with a mean age of 77.4 ± 10.2 years. Baseline patient demographics are presented in Table 1.

**TABLE 1:** Baseline clinical characteristics of patients with severe AECOPD.

	Whole cohort (*n* = 11,465)
Age (years)	77.4 ± 10.2
**Gender**	
Male	10,421 (90.9%)
Female	1044 (9.1%)
CCI	4.5 ± 1.8
Severe AECOPD in the past 12 months	430 (3.8%)
**Medical co‐morbidities**	
HT	4842 (42.2%)
DM	2004 (17.5%)
Dyslipidaemia	2394 (20.9%)
Stroke/TIA	1019 (8.9%)
Heart failure	1938 (16.9%)
Ischemic heart disease	2021 (17.6%)
Bronchiectasis	462 (4.0%)
**Medication**	
ICS	7891 (68.8%)
LABA	9593 (83.7%)
LAMA	9890 (86.3%)
Roflumilast	1633 (14.2%)
Theophylline	1920 (16.7%)
**Baseline laboratory parameters**	
BEC (x cells/µL)	452 ± 589
Serum Cr (µmol/L)	93.8 ± 60.1
Serum albumin level (g/L)	33.6 ± 10.2

*Note:* Data expressed as mean ± SD.

Abbreviations: AECOPD, acute exacerbation of chronic obstructive pulmonary disease; BEC, blood eosinophil count; CCI, Charlson comorbidity index; Cr, creatinine; DM, Diabetes mellitus; HT, Hypertension; ICS, inhaled corticosteroids; LABA, long‐acting beta‐agonist; LAMA, long‐acting anti‐muscarinic agents; TIA, transient ischaemic attack.

### 3.1 Trends From 2022 to 2024

A significant increasing trend in severe AECOPD admissions was observed over the 3‐year period (*p* value for between‐group differences = 0.003) (Figure 1). The number of patients and admission episodes to acute medical hospitals under HKHA were 4025 patients/6178 admission episodes in 2022, 6095 patients/8754 admission episodes in 2023 and 6374 patients/10,121 admission episodes in 2024, respectively.

**FIGURE 1:**
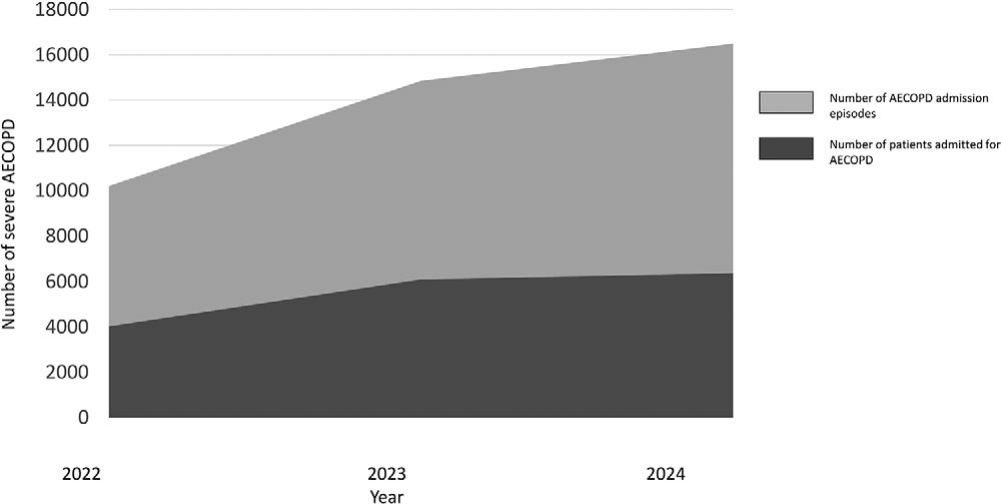
Number of headcounts admitted for severe AECOPD and number of severe. AECOPD hospital admission from year 2022 to 2024. AECOPD, acute exacerbation of chronic obstructive pulmonary disease.

### 3.2 Phenotype and Burden of AECOPD

Among the included patients, 5128 (44.7%) of them had an eosinophilic phenotype, whereas 6337 (55.3%) had a non‐eosinophilic phenotype, with the phenotype defined by baseline BEC ≥ or <300 cells/µL.

The mean annual total health‐care costs for severe AECOPD were comparable between these groups (HKD 80,136 ± 117,608 for eosinophilic phenotype vs. HKD 79,230 ± 131,450 for non‐eosinophilic phenotype; *p* = 0.70 in both univariate and multivariable linear regression) (Figure 2 and Table S1).

**FIGURE 2:**
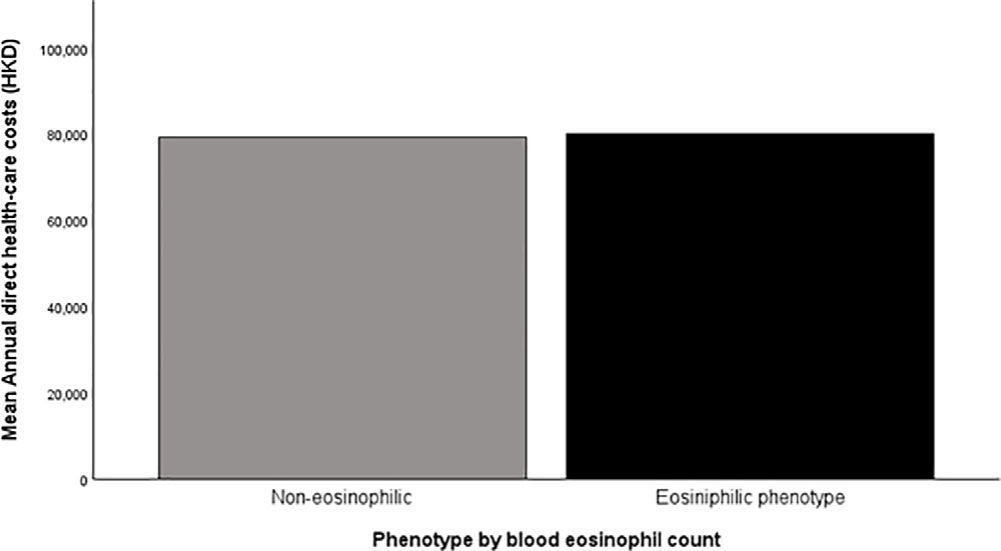
Annual total direct health‐care costs stratified by phenotype.

Patients with co‐existing bronchiectasis (*n* = 462) had a significantly higher annual number of severe AECOPD and annual direct health‐care costs from severe AECOPD than those without bronchiectasis (*n* = 11,003) (annual number of severe AECOPD: 2.16 ± 2.84 vs. 1.49 ± 1.61, *p* < 0.001; HKD 113,412 ± 145,593 vs. HKD 78,218 ± 124,332, *p* < 0.001 in both univariate and multivariable linear regression) (Table S1).

### 3.3 Annual AECOPD Frequency and Burden of AECOPD

The mean annual number of severe AECOPD was 1.52 ± 1.68 per patient. There were 1653 patients who had ≥2 severe AECOPD per year, whereas 9812 had <2 severe AECOPD per year. The mean annual total direct health‐care costs attributed to severe AECOPD were HKD 55,309 ± 84,334 and HKD 224,037 ± 206,351 for patients with fewer than 2 and at least 2 severe AECOPD per year respectively, *p* < 0.001 in both univariate and multivariable linear regression analyses (Figure 3 and Table S1). A strong positive correlation was found between the annual number of severe AECOPD with total direct health‐care costs and annual direct health‐care costs (*r* = 0.536, *p* < 0.001 and *r* = 0.612, *p* < 0.001, respectively).

**FIGURE 3:**
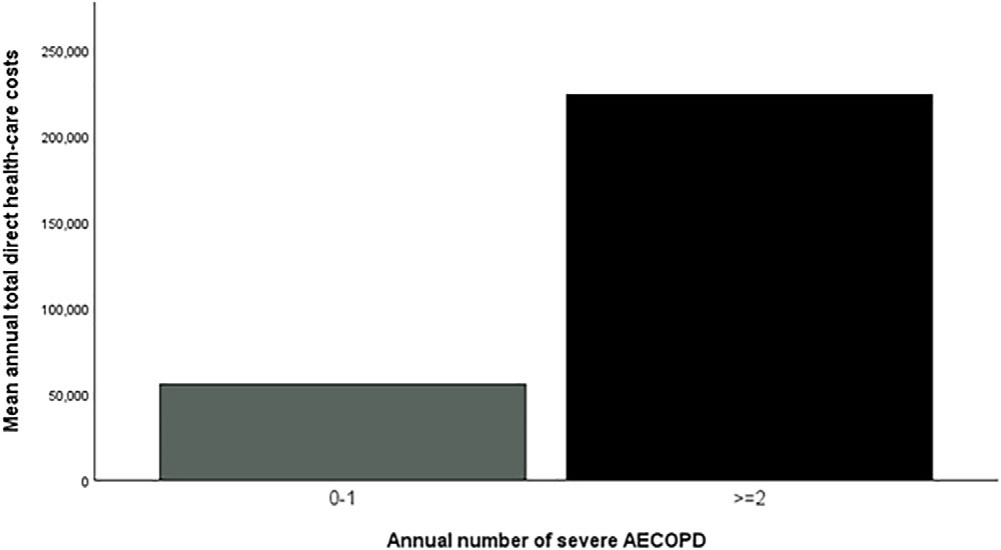
Annual total direct health‐care costs stratified by annual severe AECOPD number. AECOPD, acute exacerbation of chronic obstructive pulmonary disease.

Furthermore, the annual number of severe AECOPD in the 1‐year period before the index admission for the individual patient was significantly associated with both the subsequent annual number of severe AECOPD and its associated costs (*r* = 0.553, *p* < 0.001 and *r* = 0.355, *p* < 0.001, respectively).

## 4 Discussion

Our study highlights the considerable health‐care and health‐economic impact of severe AECOPD in Hong Kong during the post‐COVID era. Severe AECOPD continues to be a main contributor to health‐care utilization, owing to extensive hospital bed usage and significant direct costs. We observed an increase in the number of severe AECOPD cases from 2022 to 2024, indicating a post‐COVID‐19 pandemic effect that has been noted in other diseases as well [19, 20, 21]. This trend may partly reflect changes in the epidemiology of different respiratory pathogens, which influence the incidence of severe AECOPD, as bacterial and viral infections are believed to account for up to 80% of cases [22]. The increasing patient volume, hospital admissions, and associated direct health‐care costs place a substantial burden on the health‐care system.

In this study, we found that severe AECOPD significantly impacts direct health‐care costs, especially in patients experiencing more frequent AECOPD. The presence of co‐existing bronchiectasis was also associated with higher direct health‐care costs. When examining phenotypes, on the basis of BEC, both eosinophilic and non‐eosinophilic groups incurred similarly high direct health‐care expenses. These results underscore the well‐established role of severe AECOPD as a major contributor to health‐care and health economic burden [23] and align with evidence that comorbidities are important factors driving this burden [24].

Bronchiectasis was reported to have dynamic interactions with COPD, contributing to an increased disease burden. It is present in approximately 5%–10% of patients experiencing AECOPD [25]. Kim et al. reported that COPD patients with co‐existing bronchiectasis experience higher rates of exacerbations requiring antibiotics, more frequent moderate‐to‐severe exacerbations, increased treatment costs, and longer treatment durations compared to those with COPD alone [26]. Additionally, these patients tend to have longer hospital stays during exacerbations [27], although some studies have questioned the extent of bronchiectasis's negative impact on AECOPD [25].

For the eosinophilic and non‐eosinophilic groups, the direct health‐care expenses were not significantly different between both groups. Similar results were also reported in other studies. In Ko et al., which assessed the health‐care resource utilization (HCRU) and clinical characteristics of patients with COPD in Hong Kong, those with eosinophilic phenotype had more COPD exacerbations but the HCRU (including COPD‐related outpatient and inpatient costs, COPD‐related direct health‐care costs, and all‐cause direct health‐care costs) was not significantly different among the patients with eosinophilic and non‐eosinophilic phenotypes [28]. Another study in Canada also reported that the average of the total cost was not significantly different among patients with or without eosinophilia as determined by the admission blood count [29]. This can be explained by the fact that eosinophilic COPD tends to have higher AECOPD risks [30], but these patients also respond better to systemic corticosteroid treatment with shorter length of stay [31, 32, 33]. These two factors could possibly explain the comparable direct health‐care expenses in the two groups.

The significant burden of severe AECOPD underscores the urgent need for effective preventive strategies. The 2025 GOLD report [34] introduced two new treatment modalities for Group E COPD patients: dupilumab and ensifentrine [35, 36]. Additionally, GOLD emphasizes the importance of vaccinations against various bacterial and viral respiratory pathogens which could trigger AECOPD [34]. Given the substantial negative impact of AECOPD, implementing timely and appropriate prevention strategies is essential and should be prioritized.

Another significant finding from our study is that 28.5% of the patients (3624 out of 11,465) died within the study period. This high mortality rate aligns with previous research; for instance, Slenter et al. reported a 1‐year mortality rate of 28% following an AECOPD [37]. The mortality rate was even higher among patients requiring ventilatory support, reaching 45.7% and 41.8%, respectively [38]. Our findings, along with existing literature, emphasize that the risks associated with AECOPD go beyond hospital readmission and health‐care costs, encompassing a concerning mortality rate.

To conclude, severe AECOPD continues to be a significant health‐care challenge in Hong Kong, with an increasing burden in the post‐COVID‐19 era. It leads to substantial bed utilization and imposes significant economic costs through direct health‐care expenses. Moreover, the high mortality rate associated with severe AECOPD underscores the importance for clinicians to recognize and address its potentially severe consequences.

Our study has several limitations. First, we did not analyze the detailed etiological factors for each severe AECOPD episode; however, as the primary aim was to assess the overall burden, this limitation is unlikely to significantly affect our conclusions. Second, most of the included patients were Chinese and male, which may impact the generalizability of our findings, although similar results observed in previous sections suggest that ethnic differences may not be substantial The male predominance reflects the local situation which is also reported in other local studies [3, 39]. Third, spirometry parameters and symptom severity were not evaluated, but this does not detract from our focus on severe AECOPD episodes and their associated burden. Fourth, some clinical parameters were not available in CDARS, such as lung function parameters, and symptom scores. These data can provide details in assessing disease severity in COPD. However, to assess the health economic burden of COPD, these data are not considered to be crucial. Furthermore, the history of prior exacerbation can also broadly stratify the patients into Group E or non‐Group E COPD. Lastly, medication costs, outpatient costs and indirect health economic burden from income loss were not incorporated into the economic burden analysis. As this study mainly aims to assess the health‐care costs from AECOPD, these costs were not collected and presented in the current study. Nonetheless, as all patients experienced at least one severe AECOPD (classified as GOLD Group E COPD) and required at least dual long‐acting bronchodilators, the costs of these standard medications are largely comparable in Hong Kong, making their impact on health‐care cost analysis minimal. Additionally, as most patients were beyond retirement age of 65 (mean age 77.4), the income loss was considered to be negligible.

## 5 Conclusions

Severe AECOPD imposes a substantial burden on the health‐care system. The presence of bronchiectasis and a higher frequency of severe AECOPD episodes are possible factors associated with increased direct health‐care costs.

## Author Contributions

**Wang Chun Kwok**: conception and design of study, data analysis, manuscript writing. **Ali Choo**: conception and design of study, data analysis, manuscript writing. **Chun Ka Wong**: manuscript revision, data curation, supervision. **Isaac Sze Him Leung**: manuscript revision, data curation, supervision. **James Chung Man Ho**: manuscript revision, data curation, supervision. **Ting Fung Ma**: data analysis and processing. **Lu Zhou**: data analysis and processing.

## Funding

This research was supported by Sanofi Hong Kong Limited.

## Ethics Statement

The study was approved by the Institutional Review Board (IRB) of the University of Hong Kong and Hospital Authority Hong Kong West Cluster (UW 23–356).

## Consent

As it was a retrospective analysis involving de‐identified data and did not involve active patient recruitment, the IRB waived the requirement for patient informed consent. The study was carried out in accordance with the principles of the Declaration of Helsinki.

## Conflicts of Interest

The authors declare no conflicts of interest.

## Guarantor Statement

James Chung Man Ho takes responsibility for the content of the manuscript, including the data and analysis.

## Supporting information




**Supporting File**: puh270425‐sup‐0001‐SuppMat.docx

## Data Availability

All available data are presented in the manuscript, and no additional data will be provided. Due to the ethical restrictions imposed by the IRB regulations and rules on patient data privacy in this study, the supporting research data are not available upon request.
