# Supplementary material for: Health‐Care Burden of Acute Exacerbation of Chronic Obstructive Pulmonary Disease in Hong Kong in the Post‐COVID Pandemic Era—Impact of Phenotype and Exacerbation History
Source: Public Health Chall. 2026 Oct 27;5(4):e70425. doi: 10.1002/puh2.70425 (PMC13712713; doi:10.1002/puh2.70425)
Supplement: Supplementary file 1 [file PUH2-5-e70425-s001.docx]

**Supplementary Table 1** Linear regression on COPD phenotype and total healthcare costs

|  |  | **R square** | **Adjusted R square** | **Unstandardized Beta coefficient** | **Standardized Beta coefficient** | **95% CI** | **p-value** |
| --- | --- | --- | --- | --- | --- | --- | --- |
| **Eosinophilic phenotype** | Univariate analysis | 0.004 | 0.000 | 307 | 0.004 | -1233 - 1847 | 0.70 |
|  | Multivariable analysis | 0.39 | 0.39 | 899 | 0.01 | -8295 - 10092 | 0.70 |
| **Bronchiectasis** | Univariate analysis | 0.06 | 0.003 | 35194 | 0.055 | 23534 - 46854 | <0.001 |
|  | Multivariable analysis | 0.39 | 0.39 | 3286 | 0.04 | 2051 - 4523 | <0.001 |
| **At least 2 severe AECOPD per year** | Univariate analysis | 0.18 | 0.18 | 114320 | 0.42 | 109846 - 118795 | <0.001 |
|  | Multivariable analysis | 0.20 | 0.19 | 105075 | 0.39 | 100461 - 109689 | <0.001 |

Linear regression findings

Multivariable regression adjusted for age, sex, COPD medication use, severe AECOPD in the 1-year period (except for at leats 2 severe AEC and CCI

AECOPD: Acute exacerbation of chronic obstructive pulmonary disease, CI: Confidence interval
